# Development and Assessment of an E-Learning Course on Breast Imaging for Radiographers: A Stratified Randomized Controlled Trial

**DOI:** 10.2196/jmir.3344

**Published:** 2015-01-05

**Authors:** Inês C Moreira, Sandra Rua Ventura, Isabel Ramos, Pedro Pereira Rodrigues

**Affiliations:** ^1^Faculty of Medicine of the University of PortoPortoPortugal; ^2^S João Hospital CenterRadiology Department and Breast UnitPortoPortugal; ^3^School of Allied Health ScienceMorphological Sciences DepartmentPorto Polytechnic InstituteVila Nova de GaiaPortugal; ^4^School of Allied Health ScienceRadiology Department, Activity and Human Movement CenterPorto Polytechnic InstituteVila Nova de GaiaPortugal; ^5^Center for Health Technology and Services ResearchPortoPortugal; ^6^Artificial Intelligence and Decision Support LaboratoryPortoPortugal

**Keywords:** breast neoplasms, continuing education, distance learning, evaluation studies, mammography

## Abstract

**Background:**

Mammography is considered the best imaging technique for breast cancer screening, and the radiographer plays an important role in its performance. Therefore, continuing education is critical to improving the performance of these professionals and thus providing better health care services.

**Objective:**

Our goal was to develop an e-learning course on breast imaging for radiographers, assessing its efficacy, effectiveness, and user satisfaction.

**Methods:**

A stratified randomized controlled trial was performed with radiographers and radiology students who already had mammography training, using pre- and post-knowledge tests, and satisfaction questionnaires. The primary outcome was the improvement in test results (percentage of correct answers), using intention-to-treat and per-protocol analysis.

**Results:**

A total of 54 participants were assigned to the intervention (20 students plus 34 radiographers) with 53 controls (19+34). The intervention was completed by 40 participants (11+29), with 4 (2+2) discontinued interventions, and 10 (7+3) lost to follow-up. Differences in the primary outcome were found between intervention and control: 21 versus 4 percentage points (pp), *P*<.001. Stratified analysis showed effect in radiographers (23 pp vs 4 pp; *P*=.004) but was unclear in students (18 pp vs 5 pp; *P*=.098). Nonetheless, differences in students’ posttest results were found (88% vs 63%; *P*=.003), which were absent in pretest (63% vs 63%; *P*=.106). The per-protocol analysis showed a higher effect (26 pp vs 2 pp; *P*<.001), both in students (25 pp vs 3 pp; *P*=.004) and radiographers (27 pp vs 2 pp; *P*<.001). Overall, 85% were satisfied with the course, and 88% considered it successful.

**Conclusions:**

This e-learning course is effective, especially for radiographers, which highlights the need for continuing education.

## Introduction

### Overview

In Europe, breast cancer is responsible for one in every six deaths from cancer in women [1]. In Portugal, breast cancer-related mortality incidence reaches 1500 women every year [2]. Thus, early detection and diagnosis of breast cancer is essential to decrease its associated mortality rate, and mass screening is recommended by the medical community [3].

### Mammography

Mammography is currently considered the best imaging technique for breast cancer screening [3] and the most effective tool for the early detection of this disease, helping reduce mortality and increasing treatment options [4,5]. Due to its importance, not only for screening but also for diagnosis, intervention, and follow-up [6], mammography has undergone constant improvements to enhance its diagnostic quality, namely in image acquisition, equipment design and components, and technical parameters [7]. Also, other diagnostic technologies are being developed such as breast tomosynthesis, which aims to reduce or eliminate the tissue overlapping effect thus detecting lesions with higher sensitivity [8].

### Radiographer Role

Radiographers play an important role in the performance of mammographic examinations; their knowledge requirements go beyond radiation exposure, positioning techniques, and other characteristics of the equipment used [9-11].

Radiographers are part of a multidisciplinary team, both during screening or diagnosis and intervention, allowing them to be aware of the clinical information as well as previous breast exams of the patient [10,11]. Therefore, it is important that the radiographer establishes direct contact with the radiologist in order to contribute to a proper diagnosis. Together with the radiologist, the radiographer is responsible for image quality assurance; the availability, accessibility, and interpretation of mammographic images; and for the performance of additional imaging, preoperative localization, and biopsy techniques [9,10].

Beyond these technical issues, radiographers are usually the first professionals to have face-to-face contact with women in primary health care, during breast cancer screening. Therefore, they should be able to provide the patient with sound answers regarding the examination and the implications of the results [11]. According to the European Society of Breast Cancer Statistics (EUSOMA) recommendations [9,10], the correct understanding of senology concepts such as breast cancer statistics and family history, and knowledge of breast disease symptoms and treatment options are critical. Further, radiographers require a fair degree of sensitivity since mammography causes considerable anxiety in most women [11].

This extended role could contribute to decreasing the mortality rate of breast cancer and thus reducing health care needs [5,11].

In this context, the knowledge of senology concepts is essential to the health professional, highly considered in some countries (such as in England, Denmark, Australia, and United States) [11-14]. Unlike the aforementioned countries, breast cancer screening in Portugal is not fully organized by the government but by a private institution [2]. This may be one of the reasons why there are no existing specific training programs and the role of the radiographer is not as differentiated and valued as elsewhere. Hence, the need for continuing education for Portuguese radiographers in this area is critical.

### E-Learning in Health Care

Continuing education is known to improve the performance of professionals, providing better health care services [15]. For example, the National Health Service University identified e-learning as a central strategic delivery mechanism for all its professionals [16]. Several studies [12,17,18] have shown that radiographers are receptive to new technologies and training and are able to upgrade their skills and extend their role.

The asynchronous ability, cost-savings, personalized learning, increased accessibility, ease of distribution, and updated content are some examples of e-learning advantages [19,20]. However, time constraints and the difficulty of “ease of use” are commonly pointed out as drawbacks [16].

With the development of new information technology, there is a surplus of software that can be used to implement e-learning systems, ranging from websites and email to blogs, wikis, and discussion forums [19]. Dedicated Learning Management Systems (LMS) support the planning, organization, and access control of specific learning processes. In radiology, there are some LMS technologies that improve collaboration, interactivity, simulation, and self-testing [20]. E-learning is therefore a useful tool helping not only students but also professionals move towards a vision of lifelong and continuous education [19,20].

### E-Learning Evaluation

The potential of e-learning may not always translate into significant improvements in educational outcomes [21]. For this reason, e-learning needs to be justified by its effectiveness and relevance [22]. One important method for evaluating a learning system is the framework developed by Kirkpatrick [23], which consists of four categories: learner perception, knowledge, behavior, and impact on organization.

Most of the studies on the evaluation of e-learning processes rely on users’ satisfaction and knowledge [22-25]. Although some authors believe that there is no evidence that learners learn more from e-learning than traditional learning, it is acknowledged that they can learn more effectively [21,22]. E-learning combined with traditional learning—that is, blended learning, or b-learning—is considered the best way to obtain higher knowledge gain [21-23]. Using pre- and post-knowledge tests within an experimental study, if the experimental group performs better than the control group, the e-learning system can be identified as the cause of the improvement [23]. According to certain authors [22,26], there is a lack of randomized controlled trials (RCTs) in research on e-learning, despite RCTs being considered the best way to assess e-learning efficacy and effectiveness. Therefore, RCTs are recommended for evaluating e-learning systems [21,22,26].

One of the most cited questionnaires for user satisfaction with e-learning [27] considers four dimensions of the e-learner satisfaction measurement: content, learner interface, personalization, and learning community. From these dimensions, the tool specifies 26 items using a 7-point Likert scale (ranging from “strongly disagree” to “strongly agree”), although the last two questions actually reflect global measures related to overall satisfaction and overall success of the e-learning system. Globally, the questionnaire has presented reliability (Cronbach alpha) of .95 [27].

The work presented in the following sections aims to provide a new easy-to-use e-learning course, thus contributing to senology teaching, while emphasizing continuing education and professional development.

### Objectives

In this context, our research question is whether an e-learning system improves the senology knowledge of radiographers and radiology students. The objectives of this work include knowledge promotion and understanding of all aspects of breast illness and patient care required by radiographers. To achieve this, we proposed to (1) develop an easy-to-use course in e-learning environment, (2) assess its efficacy and effectiveness, and (3) assess the satisfaction of users.

## Methods

### Overview

An asynchronous e-learning course on breast imaging for radiographers was developed and evaluated for its efficacy, effectiveness, and satisfaction.

### Target Population and Sample Strategy

The target population in this study was all radiographers working at public health institutions in Porto’s metropolitan area, in Portugal, with ability to perform mammographic exams, and all radiography students attending the 3^rd^ and 4^th^ years of the radiology course at the School of Allied Health Science, Porto Polytechnic Institute who already had mammography training.

Hence the sample was stratified by professional status into two groups: students and radiographers, who were invited by email to participate in the study, after an individual request.

### Randomization

All radiographers and students included in the study were asked to perform the pre-knowledge test. Randomization was performed with the population elements in each stratum who accepted and effectively took the pretest. Therefore, 50% of the elements of each stratum were randomly allocated to the intervention group and contacted to participate in the course, after being properly informed of the randomization process. The remaining sample was allocated to the control group and afterward contacted to participate in a second test.

### Implementation

**Figure 1:**
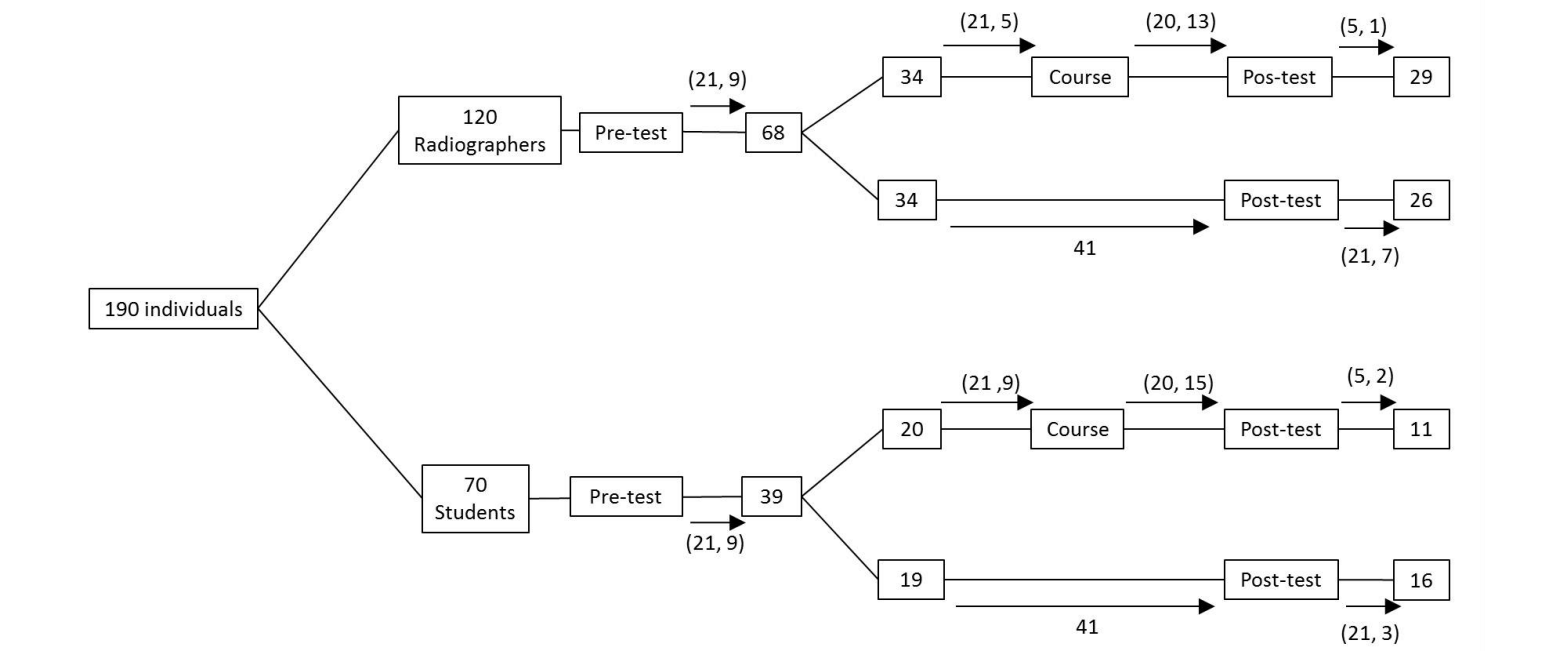
Study design and post-implementation duration: numbers in boxes represent number of participants; numbers in arrows correspond to the elapsed time in days (maximum, average) at each phase.

The communication with all participants was performed mainly by email (maximum three attempts) as described in Figure 1.

The intervention group was contacted in order to perform the e-learning course, and a similar process to the pretest enrollment occurred. Private asynchronous access was given during a 20-day period to those who agreed to do the course. Then, a final assessment test was delivered, to be answered within 5 days. Those who discontinued the intervention were requested nonetheless to perform the posttest, to allow an intention-to-treat analysis.

The control group was contacted 41 days after randomization to perform another test. During this period, neither radiographers nor students allocated to the control group had any formal educational activity concerning senology lectures given by our research team and were asked to answer tests in an honest manner, without consulting external sources, which could in the future evolve into a clear accreditation process [28].

The course was created and revised between October 2011 and January 2012. Contact with the participants occurred between February 1 and March 7, 2012, and the trial was conducted between March 7 and May 31, 2012.

### The E-Learning Course Description

The course was written in Portuguese and developed in Netbeans version 8.0, using simple and wide-spread technologies such as Hypertext Preprocessor (PHP), HyperText Markup Language (HTML), JavaScript, Cascading Style Sheets (CSS), and Extensible Markup Language (XML), and was hosted on the server at the Faculty of Medicine of the University of Porto. Website security was guaranteed through an authentication mechanism with username and password, and no collaborative activities [29] were available, setting the focus on self-learning.

The course instructions were available on the website, along with a glossary, and it was structured into four modules (Table 1). The contents were based on guidelines proposed by EUSOMA [9].

**Table 1:** Description of the main contents included in the intervention e-learning course.

Module	Contents
1. Breast anatomy and physiology of breast	Breast localization and superficial anatomy
	Breast tissues constitution
	Radiological anatomy
	Breast tissues patterns
	Breast lesions localization
2. Breast cancer: multidisciplinary approach	Breast cancer statistics
	Breast cancer screening: Mammography
	Breast cancer signs and symptoms
	BI-RADS classification
	Additional imaging techniques
	Breast cancer management and treatment options
3. Breast pathology	Pathology of benign and malignant lesions
	Male breast cancer
4. Mammography: technical approach	Mammography history
	Technical aspects of the equipment and new technologies
	Image quality control
	Positioning techniques and indications for standard and additional mammographic views
	Localization and biopsy techniques for non-palpable lesions
	Radiographer role

**Figure 2:**
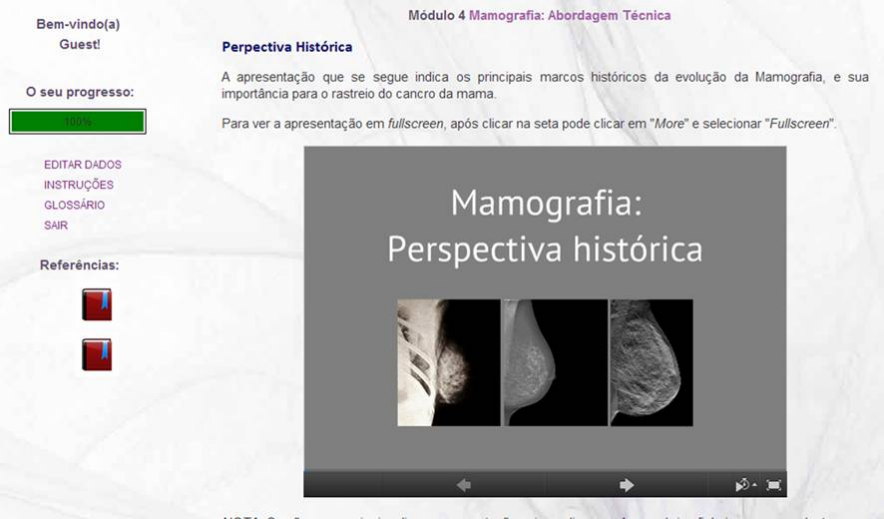
Screenshot of a Prezi presentation in the e-learning course.

**Figure 3:**
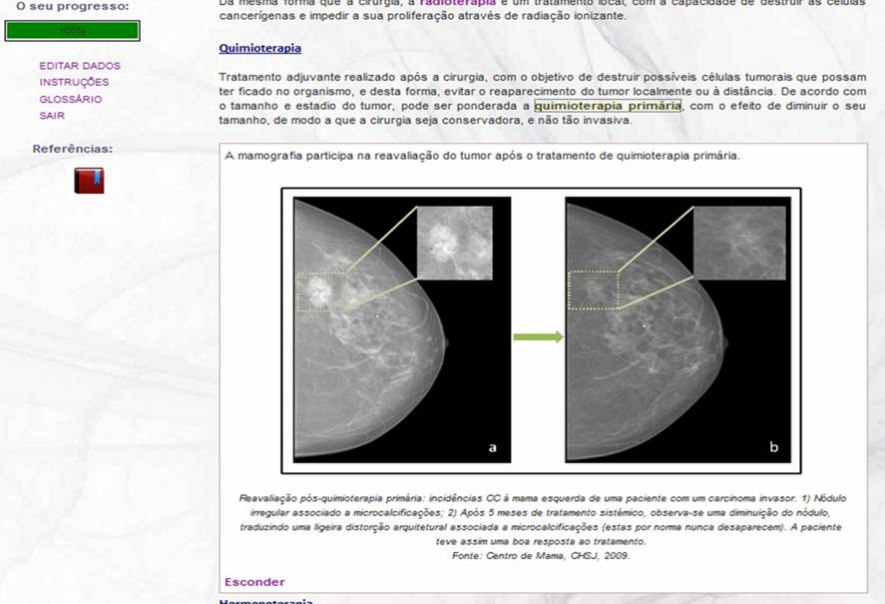
Screenshot of mammography views in the e-learning course.

**Figure 4:**
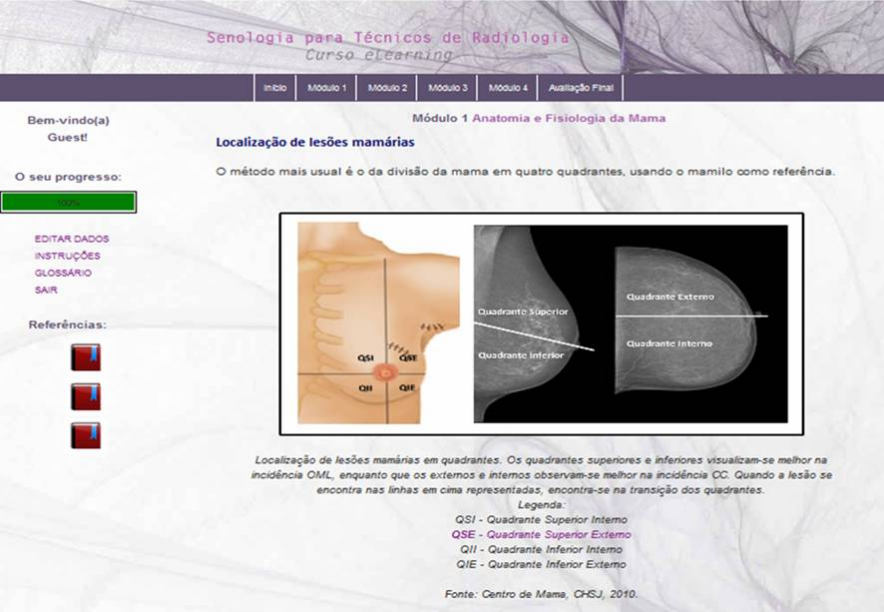
Screenshot of mammography views and schemes in the e-learning course.

**Figure 5:**
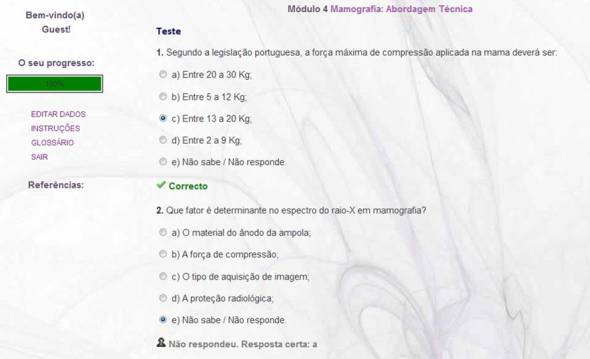
Screenshot of one of the four self-assessment tests included in the e-learning course.

The content was presented using text, images, videos, and Prezi presentations. Diagnostic images were collected directly from the Breast Unit of S João, Hospital Center, with proper legal authorization. All content was reviewed by specialists from the same institution. Screenshots are presented in Figures 2-5.

Given the asynchronous characteristics of the course, learners could monitor the evolution of their learning through a status bar, giving feedback about the self-learning. At the end of each module, a summary of the key points and a self-assessment test of 6 multiple-choice questions were presented; correct answers were immediately available. The posttest was administered at the completion of the course. This posttest was a final assessment sent by email and available for 5 days after mail delivery. After successful completion, a certificate of attendance was sent to the participants.

### Pre- and Post-Knowledge Tests

Each test was structured with 8 multiple-choice questions related to the course modules (Table 1). Questions from pre- and post-knowledge tests were different but had the same level of difficulty. To ensure this condition, a pilot study was done with a convenience sample of 8 radiographers not included in the final sample.

Participants were asked to answer in an honest manner, without consulting external sources. This study consisted of two phases. In the first step, 4 individuals received one test, and the other 4 received the other test. The random delivery of the tests was accomplished, highlighting the independence of the questionnaires in relation to being the first or the second test answered. Then, in a second step, when an individual submitted the answers 24 hours later, the other test was sent. This pilot study was done between February 18 and March 1, 2012.

The focus of the data analysis was to determine whether there was a significant difference between the questions presented in the first and the second tests. For this observation, we used paired Wilcoxon tests to compare the number of incorrect answers of each individual between the first and the second test. As the questions were organized by themes, related to the modules of the course, the tests were also organized for each set of questions. The statistical analysis was performed using IBM SPSS Statistics software version 17.0, and the significance level was .05.

Through the data analysis, we can conclude that there are no differences among the modules in one test and the other: module 1 (*P*=.083), module 2 (*P*=.096), module 3 (*P*=1.000), and module 4 (*P*=.317). Apart from the non-existence of differences, some questions and multiple choices were readjusted in order to make them clearer.

Demographic information such as age, gender, academic qualifications, years of professional experience, and routine mammography, as well as opinions regarding the need for continuing education and the receptivity of e-learning programs on any topic of professional interest, were also gathered.

The Web resource GoogleDocs was used to create and deliver online the tests, assigned to individuals randomly as pre- or post-knowledge tests.

### Satisfaction Questionnaire

Given the lack, to the best of our knowledge, of a questionnaire in Portuguese, we used the questionnaire proposed by Wang [28], after translation by an expert bilingual translator.

### Assessment of Outcomes

The primary outcome was the knowledge evolution observed between pre- and post-knowledge tests (each measured by the percentage of correct answers and failure defined using 50% cut-off), using a paired analysis the difference between pre- and post-knowledge test percentage of correct answers (referred to as “evolution” and measured in percentage points [pp]).

Effectiveness was assessed through the proportion of participants who completed the intervention. Satisfaction was mainly assessed through the last two items of the questionnaire.

### Statistical Analysis

Analysis was mostly performed according to the intention-to-treat strategy [30]. Normality was tested with the Kolmogorov-Smirnov test (total sample) and the Shapiro-Wilk test (for each group), beyond the visual analysis of histograms.

The sample was described by average (µ) and 95% confidence intervals for normally distributed variables, and median, 25 and 75 percentiles (P_25_; P_75_) for the remaining.

Homogeneity between the two groups was assessed using the Mann-Whitney *U* test. Differences in the outcome of the two groups were assessed using the Student’s *t* test. Chi-square test or Fisher’s exact test were used to examine the association between nominal variables.

We considered a significance level of .05, and the analysis was carried out in IBM SPSS Statistics software, version 17.0.

## Results

### Summary

**Figure 6:**
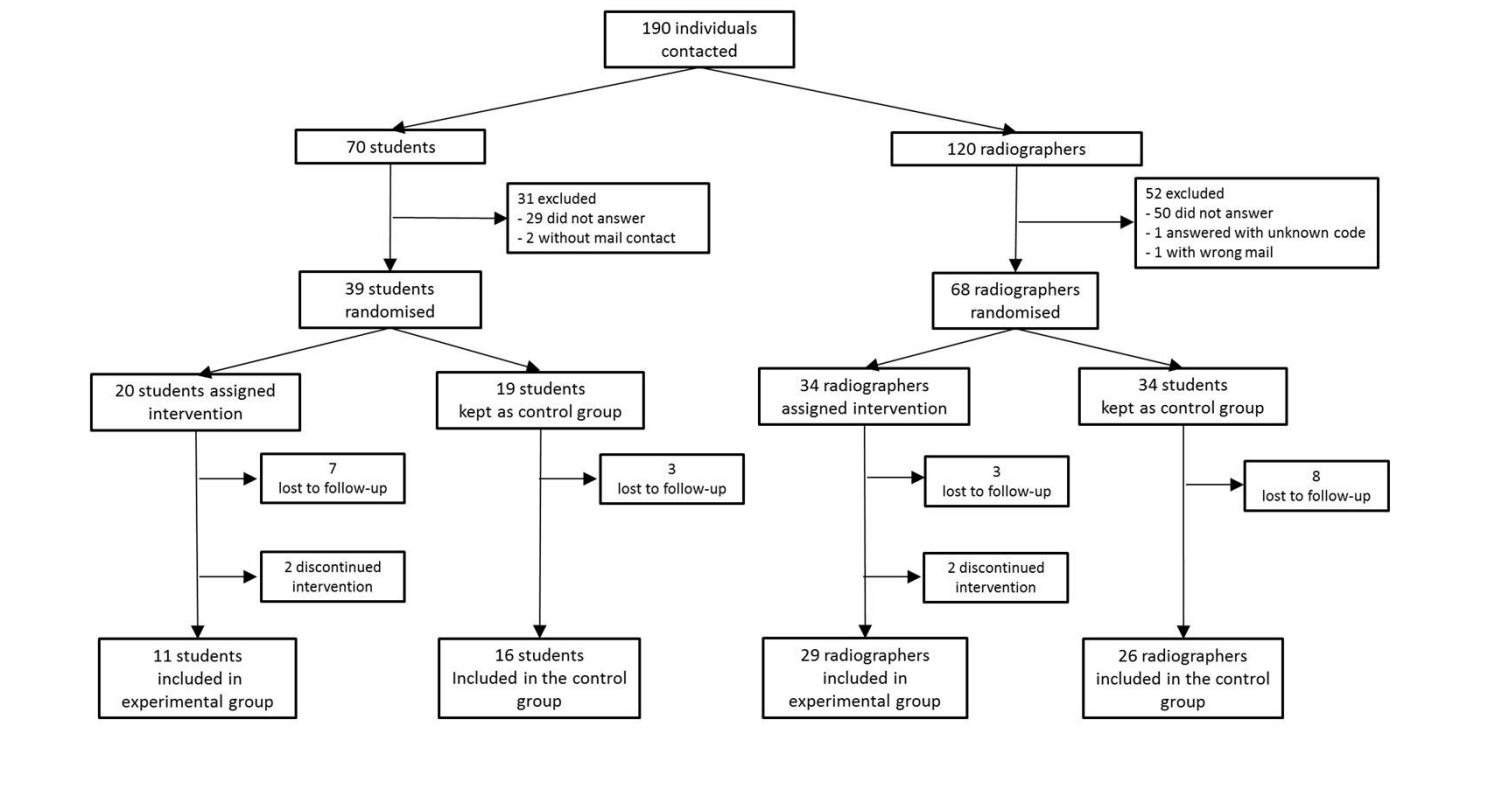
Participant flow diagram showing enrolled sample and respective dropouts. Individuals who did not answer the participation request were considered "lost to follow-up"; participants who did not finish the course were considered “discontinued intervention”.

Globally, 190 individuals were considered for inclusion (120 radiographers and 70 students) from which a total of 107 enrolled and answered the pre-knowledge test (68 radiographers and 39 students). The average time spent (in days) between study’s milestones can be observed in Figure 1. Likewise, the participants’ flow including dropouts (“lost to follow-up” and “discontinued interventions”) is shown in Figure 6.

According to the intention-to treat strategy, the intervention group included 2 radiographers and 2 students identified as lost to follow-up, as well as 1 radiographer and 1 student identified as “discontinued intervention” who answered the post-knowledge test. Globally, 46 individuals were included in the intervention group (14 students and 32 radiographers) and 42 in the control group (16 students and 26 radiographers).

### Sample Description

Of the 107 participants, 36.4% (39/107) were students and 63.6% (68/107) were radiographers; 79.4% (85/107) were female. The median age was 21 years old (P_25_=21; P_75_=22) for students, and 33 years old (28; 40) for radiographers. Overall, 10.3% (11/107) were third-year students, 26.2% (28/107) were fourth-year students, 1.0% (1/107) was credited due to professional experience, 7.5% (8/107) had bachelor’s degrees, 50.5% (54/107) had graduated, and 4.7% (5/107) had a master’s degree.

In the radiographer group, the median professional experience was 12 years (5; 17); 31% (21/68) individuals did not perform mammography at all, 53% (36/68) performed fewer than 30 per week, 6% (4/68) performed between 30 and 40 per week, and 10% (7/68) performed more than 40 per week.

In the pre-knowledge test, there was a failure rate of 14.0% (15/107). We observed that 51.4% (55/107) of the results were between 50-75% and 34.6% (37/107) had results better than 75%. In the post-knowledge test, the failure rate was 5.6% (6/107); 25.2% (27/107) of the results were between 50-75%, and 51.4% (55/107) had results better than 75%.

Intervention and control groups were globally comparable, with a difference only in female proportion in both groups for the radiographer stratum (88% vs 68%, *P*=.041).

### Efficacy

Both intervention and control groups had similar results in the pre-knowledge test (63% vs 63%, *P*=.159), with also no differences per stratum (63% vs 63%, *P*=.626).

The intervention group obtained better results in the post-knowledge test compared to the control group (88% vs 63%, *P*<.001), and no differences were found between the two strata (75% vs 75%, *P*=.261).

Participants had an overall positive evolution (μ=13 pp, 95% CI 8-18), which is higher in the intervention group (21 pp vs 4 pp, *P*<.001) but similar between students and radiographers (11 pp vs 14 pp, *P*=.601). Furthermore, the control group had an inconclusive evolution (95% CI -3 to 11).

**Figure 7:**
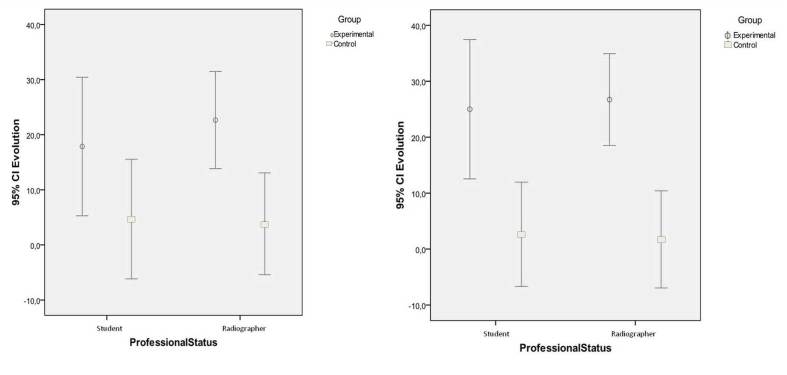
Improvement stratified by professional status. Left plot (intention-to-treat): Inconclusive result for students but clear effect for radiographers. Right plot (per-protocol): Clear effect for both students and radiographers.

Stratifying the results (left plot of Figure 7), the difference in the evolution of students, although favorable to the intervention group is not statistically significant (18 pp vs 5 pp, *P*=.098), while in the radiographers, the effect of the course is clear (23 pp vs 4 pp, *P*=.004). There were no differences in the evolution between students and radiographers both in the intervention group (18 pp vs 23 pp, *P*=.531) and in the control group (5 pp vs 4 pp, *P*=.905).

Considering a per-protocol analysis (right plot of Figure 7), those who were considered “lost to follow up” (n=10) and “discontinued intervention” (n=4) in the intervention group were allocated to the control group. As a result of this strategy, the overall evolution was found positively different in the two groups (26 pp vs 2 pp, *P*<.001).

Significant differences in students could then be observed between intervention and control (25 pp vs 3 pp, *P*=.004), along with a reinforced difference in radiographers (27 pp vs 2 pp, *P*<.001).

### Effectiveness and Satisfaction

Most participants (81%, 44/54) in the intervention group agreed to take the course (13 students and 31 radiographers), and only 9% (4/44) did not attend the full course: 2 students and 2 radiographers.

All participants who completed the e-learning course answered the satisfaction questionnaire (n=40). Considering global measures, 85% were satisfied with the e-learning system (students vs radiographers: *P*=.570) and 88% considered the system successful (*P*=.660). Detailed results were described in [31].

Of all e-learners, 10% (4/40) had previous e-learning experience, and 5% (2/40) performed it in the health area. However, the overall satisfaction did not differ between these participants and those who had no previous experience of e-learning (*P*=.191).

## Discussion

### Principal Findings

Comparing students and radiographers, we did not find any significant differences besides age and academic qualifications, and therefore no additional confounding factors were considered for adjustment. Overall improvement was observed and attributed to the course.

Although the effect was not clear in students, we found differences in post-knowledge test results between the intervention and control groups, whereas such differences were absent in the pre-knowledge test. This result could be explained by the smaller sample size or higher proportion of individuals lost to follow-up, which could have resulted from being enrolled in other learning activities at the same time during the academic year. In addition, students were probably more prone to self-learning from other sources, or there was interest from students in the control group in learning more after performing the pre-knowledge test.

Regarding radiographers, those who were allocated to the intervention group significantly improved more than the controls did, which supports the importance of continuing education throughout their working lives.

The per-protocol analysis enhanced the influence of the course, exposing a significant effect on the students’ stratum. Considering that the real course effect is probably in between the two statistical analyses, we can conclude that this randomized controlled trial showed that the e-learning course improved the knowledge of those who attended (even if only part of) the intervention.

Overall, the developed course is efficacious, especially for radiographers, which highlights the need for continuing education, foreseeing also e-learning as an increasingly viable complement to the traditional method, especially since the technologies involved do not need heavy hosting requirements. This study also proved that the course is effective, since only 10% of the learners dropped out. Moreover, the course showed to have a high level of satisfaction, for both radiographers and students.

### Limitations

Since it was not possible to find in the literature any satisfaction questionnaire for e-learning systems in Portuguese, the validity of this satisfaction evaluation should be carefully considered, given the translation. A validation of the questionnaire used in this work could be a future project.

Another limitation of this work is the single learning institute with moderate sample size, affecting the generalizability of the results.

Another restraint is related to the learning and evaluation strategy, since we faced the risk of individuals resorting to external sources in order to provide correct answers to the tests. This situation creates a slight bias that is difficult to control considering the study design.

Nevertheless, randomization was performed after completing the pre-knowledge test, which yielded that the same willingness of participants to enter the study was demonstrated, regardless of the group where they were allocated.

### Conclusions

Globally, this study underlines the importance of the radiographer as the health care professional who interacts first with women during the breast cancer screening process. We consider the high rate of participation an important aspect in our study (57% of radiographers and 56% of students), which reflects the great interest shown by these professionals to participate in scientific research, thereby promoting their professional category. They took advantage of learning opportunities, which shows that they are health care professionals committed to responding to the constant challenges of the profession.

This study contributes to the Portuguese radiographers’ continuing education, since we did not find any similar course related to breast imaging. It would be interesting to conduct additional assessments to demonstrate effective consolidation of knowledge gain. Future developments may include collaborative activities; for this first assessment, we believe such activities would confound the efficacy results.

Our main finding illustrates the knowledge improvement in senology that our e-learning course gave radiographers. We believe that this study highlights the importance of e-learning as a training platform, especially in light of budget constraints associated with the current economic climate. E-learning should be considered for continuing education, and directors should invest in it to improve the skills of their professionals and consequently enhance health care services [32,33].

## Abbreviations

EUSOMA: European Society of Breast Cancer Statistics
LMS: Learning Management Systems
pp: percentage points
RCT: randomized controlled trial
